# The Role of Adolescent Nutrition and Physical Activity in the Prediction of Verbal Intelligence during Early Adulthood: A Genetically Informed Analysis of Twin Pairs

**DOI:** 10.3390/ijerph120100385

**Published:** 2015-01-05

**Authors:** Dylan B. Jackson, Kevin M. Beaver

**Affiliations:** 1 Department of Criminal Justice, College of Public Policy, University of Texas at San Antonio, 501 W. Cesar Chavez Blvd., San Antonio, TX 78207, USA; 2 College of Criminology and Criminal Justice, Florida State University, 112 S. Copeland St., Tallahassee, FL 32306, USA; E-Mail: kbeaver@fsu.edu; 3 Center for Social and Humanities Research, King Abdulaziz University, Jeddah, Saudi Arabia

**Keywords:** nutrition, diet, physical activity, exercise, verbal intelligence, heritability, twins, DF (Defries-Fulker) analysis

## Abstract

A large body of research has revealed that nutrition and physical activity influence brain functioning at various stages of the life course. Nevertheless, very few studies have explored whether diet and exercise influence verbal intelligence as youth transition from adolescence into young adulthood. Even fewer studies have explored the link between these health behaviors and verbal intelligence while accounting for genetic and environmental factors that are shared between siblings. Employing data from the National Longitudinal Study of Adolescent Health, the current study uses a sample of same-sex twin pairs to test whether youth who engage in poorer fitness and nutritional practices are significantly more likely to exhibit reduced verbal intelligence during young adulthood. The results suggests that, independent of the effects of genetic and shared environmental factors, a number of nutritional and exercise factors during adolescence influence verbal intelligence during adulthood. Limitations are noted and suggestions for future research are outlined.

## 1. Introduction

The health of the brain and the health of the body are inextricably intertwined [1,2]. A growing number of studies have linked various aspects of cognitive functioning to behaviors that promote and maintain physical health, including adequate physical exercise [1,3,4] and nutrition [2,5,6]. Research suggests that aerobic exercise in particular facilitates the development of executive functions and language abilities in children [4,7] and a host of other cognitive functions in young, middle-aged, and older adults [3,8]. A sedentary lifestyle, on the contrary, increases the likelihood of verbal, motor, and intellectual impairment [9,10]. Nutritional factors seem to play an equally important role in neurocognitive health. A healthy diet, for instance, appears to optimize cognitive performance on verbal, visuospatial, and memory tasks among various age groups [11,12,13]. Individuals who are exposed to poor and/or inadequate nutrition, however, are at risk of exhibiting deficits in neurocognitive functioning [14,15]. Thus, it appears that efforts to sustain healthy eating and exercise behaviors correspond to enhanced brain health across the lifespan.

Although studies have linked nutritional and fitness factors to numerous cognitive functions, only a small number of these studies have examined adolescent samples [11,16]. As Hoyland and colleagues [17] noted in their review of the literature, research examining the influence of eating and exercise habits during adolescence on subsequent cognitive functioning is particularly sparse. The literature has instead focused almost entirely on the benefits of physical activity and nutrition for brain health during childhood [4,6,15] and late adulthood [1,18,19]. Nevertheless, recent research has suggested healthy eating and exercise habits during the teenage years can improve concentration, as well as verbal and reasoning ability [20]. Scholars have also generally overlooked the possibility that genetic factors might confound the link between health behaviors (e.g., nutrition, physical activity) and various cognitive functions. In an effort to address these limitations in the literature, the current study utilizes a sample of twins to explore whether: (a) nutritional and exercise factors during adolescence are predictive of verbal intelligence during early adulthood and (b) such effects remain significant after accounting for shared genetic and environmental factors.

### 1.1. The Link between Nutrition and Verbal Intelligence

Both the sufficiency and the quality of nutrition can influence cognitive functioning at various life stages [5,14,15,21]. A number of studies have indicated that foods rich in antioxidants, long-chain fatty acids, and other essential vitamins support brain functioning in multiple ways, including, but not limited to, the synthesis of neurotransmitters [22], the reduction of metal ions [22], and the production of brain-derived neurotrophic factor [2]. One aspect of neurocognitive functioning that has been repeatedly emphasized in the literature is verbal intelligence [13,14].

Researchers have paid particular attention to early childhood nutrition as a predictor of verbal intelligence [6,13,23], in large part due to the particular sensitivity of the brain during childhood [24]. In general, studies examining the influence of childhood nutrition on verbal intelligence reveal significant associations, both cross-sectionally [14,17,25] and longitudinally [5,12,26]. To illustrate, a recent study by Liu and colleagues [25] indicated that kindergarten children who ate breakfast regularly scored higher on tests of verbal intelligence than children who only ate breakfast sporadically. Studies using more comprehensive measures of diet quality appear to yield analogous results. For instance, research has found that toddlers with higher scores on a comprehensive eating assessment (*i.e.*, toddlers with healthier eating patterns) score higher on a picture vocabulary test (*i.e.*, PPVT) at age 10 [13]. Similar results were garnered by Gale *et al.* [12], who reported that healthier dietary habits during the first year of life, including higher fruit and vegetable consumption, were predictive of verbal intelligence at age 4.

Despite a general emphasis on infant and child nutrition, a number of studies employing adult samples have also found significant associations between diet quality and verbal fluency/comprehension [21,27]. For instance, Péneau and colleagues [21] studied a sample of 2533 French adults, aged 45–60 at baseline, and found the regular intake of fruits and vegetables, especially those rich in vitamins C and E, to be significantly associated with verbal memory 13 years later. Another recent study found that, even after accounting for SES and child IQ, elderly subjects who consumed a “Mediterranean” diet (*i.e.*, greater consumption of vegetables, beans, oil/vinegar dressings, fish, and poultry) were more likely to evince higher verbal intelligence than subjects with other dietary patterns [27]. Thus, it appears that nutritional factors can have long-lasting effects on various dimensions of verbal intelligence, including fluency and comprehension.

### 1.2. The Link between Physical Activity and Verbal Intelligence

In addition to the literature linking nutrition and verbal intelligence, significant associations between indicators of physical activity and verbal intelligence have also been detected [1,3,4,8,10,19]. In general, this body of research mirrors the findings of the nutrition literature: participation in regular exercise is associated with greater verbal aptitude. Again, most of the research examines child or adult samples and generally neglects adolescents. To illustrate, a study by Scudder and colleagues [4] revealed that children at a higher physical fitness level exhibited heightened processing of semantic information and sensitivity to syntactic violations when reading sentences. The results suggest that adequate physical fitness can have important implications for developing better language skills, including a more comprehensive vocabulary. A recent meta-analysis of 59 studies from 1947–2009 revealed that physical activity during childhood has a significant, positive effect on various cognitive functions, including reading and language ability [28].

Studies exploring the link between physical activity and verbal intelligence during middle and late adulthood detect a similar pattern [1,3,10,19]. A number of these studies suggest that, even after a relatively short intervention period, regular aerobic exercise can lead to improvements in verbal intelligence [3,19]. For example, a recent study found that, following a 12-week intervention involving regular participation in a “Spinning” exercise group, previously sedentary adults displayed significant improvements in semantic verbal fluency relative to controls (15% *vs*. 2% increase) [3].

Long-term exercise patterns also seem to influence verbal ability. A study by Benedict and colleagues [1] used brain imagining technology and self-report methodology to test the relationship between exercise habits and various cognitive skills in older adults. The results indicate that adults who engaged in a higher number of 30-min exercise sessions per week exhibited a larger brain volume, high white matter density, and greater verbal fluency. Despite the research highlighting the association between physical activity and verbal intelligence, studies comparing the effects of exercise on verbal ability across adults of different ages are lacking. Still, a recent study of a large sample of adults explored the link between physical activity and fluid cognitive ability across age groups and found that the youngest group (aged 20–24) derived the most cognitive benefits from exercise [8]. When it comes to the link between adult exercise and cognition, therefore, it seems that earlier involvement in physical activity is preferable.

### 1.3. The Current Study

Whether examining physical activity or dietary patterns, very few researchers have studied the influence of adolescent health behaviors on verbal intelligence, but see [11,16,20]. The handful of studies that have examined this relationship at this stage of the life course, however, suggest that nutrition and exercise still play a significant role in verbal capacity [11,16]. In addition to the paucity of literature using adolescent samples, virtually all of the research to date that has examined the interconnections between diet, physical activity, and verbal intelligence has failed to use a research design that is genetically informative, but see [29]. Although research by Luciano and colleagues [29] revealed that diet, exercise and various components of intelligence are influenced by similar genetic factors, researchers have yet to test the extent to which diet and physical exercise have an environmentally-mediated effects that are *independent* of genetic and familial/shared environmental factors. In an effort to expand upon the findings linking health behaviors to verbal ability, the current study employs a sample of adolescent twin pairs to test whether differences in their diet and exercise habits predict differences in their verbal intelligence during early adulthood, controlling for shared environmental and genetic influences.

## 2. Method

### 2.1. Sample

Data for the current study come from the National Longitudinal Study of Adolescent Health (Add Health). The Add Health is a large, nationally representative study of American youth [30]. The first wave of data collection began in the 1994–1995 school year and the most recent wave of data collection occurred in 2008. In total, four waves of data have been collected, covering approximately 14 years of development across adolescence and adulthood. At the first wave of data collection, over 90,000 youth participated in surveys that included questions about an array of behaviors as well as family and peer relationships. Additional details about a subsample of youth were garnered through parent and youth interviews, as well as through direct assessment of their cognitive abilities (e.g., language, memory). Specifically, 20,745 adolescents and 17,700 of their primary caregivers were interviewed at wave 1.

The Add Health sample is particularly useful for the agenda of the current study, as it contains a large number of siblings. At the first wave of data collection, the interviewed subsample of youth were asked whether they currently lived with co-twin, full siblings, half sibling, or cousin. If the youth answered in the affirmative, and their sibling was between the ages of 11 and 20, then their sibling was recruited for the study. Moreover, a probability sample of additional pairs of full siblings are also included within the Add Health data [31], resulting in a total subsample of approximately 5500 siblings at wave 1. Research to date suggests that this subsample resembles the full adolescent sample on a number of behavioral and demographic indicators [32,33], intimating the utility and generalizability of the sibling subsample. For reasons to be outlined, the current study utilizes a subsample of monozygotic (MZ) and same-sex dizygotic (DZ) twins who participated in waves 2 and 3 of the data collection. Although 490 twin pairs (or 980 twins) were initially eligible for inclusion, some twin pairs were of undetermined zygosity and/or were missing data, resulting in final analytical samples that ranged from N = 644 twins to N = 694 twins.

### 2.2. Measures

#### 2.2.1. Outcome Measure

*Verbal Intelligence.* At wave 3 of data collection, when the majority of respondents were between the ages of 18 and 24, Add Health researchers measured the verbal abilities of each subject by employing an abridged version of the commonly used Peabody Picture Vocabulary Test (PPVT) known as the Picture Vocabulary Test (PVT). The assessment involved a process in which the interviewer would read a word aloud while showing four distinct illustrations to the participant. Subjects were asked to select the illustration that most accurately reflected the meaning of the word for additional information on the PVT, see [34].

Importantly, the reliability and validity of the PPVT as a measure of verbal intelligence has been buttressed by prior research [35]. Scores on the PPVT are also highly correlated with scores on other cognitive batteries [36,37], which bolsters the construct validity of the measure. Included in the Add Health data are the wave 3 PVT percentile scores, which provide an indication of each subject’s verbal intelligence by calculating the percentage of subjects in the full Add Health sample with lower scores on the PVT at wave 3. We chose to utilize these percentile scores in the current study, as they provide a clear indication, in intuitive measurement units, of the verbal intelligence of each participant at wave 3. Table 1 includes the descriptive statistics of the verbal intelligence measure as well as all other variables and scales relevant to the analysis.

**Table 1:** Descriptive statistics of the MZ and same-sex DZ twin subsample.

Variable	Mean	Standard Deviation	Range
Verbal Intelligence (W3)	46.32	29.55	0–100
Fast Food Consumption	2.27	1.91	0–7
Low Vegetable Consumption	0.25	0.43	0–1
Meal Deprivation	0.24	0.23	0–1
Low Sports Involvement	1.75	1.09	0–3
Low Cycling/Skating	2.41	0.92	0–3
Low General Exercise	1.52	1.05	0–3
Insufficient Exercise	0.38	0.49	0–1
Age (W2)	17.06	1.60	13.28–20.48
Age (W3)	21.91	1.64	18–26
Sex (Male = 1)	0.52	0.50	0–1
Twin Status (MZ = 1)	0.53	0.50	0–1

#### 2.2.2. Nutrition Measures

*Fast Food Consumption.* We tapped both food quality and food sufficiency by creating three measures of nutritional inadequacy (*i.e.*, nutritional risk factors) based on several items from the second wave of data collection, when the vast majority of subjects were still adolescents. The first of these items concerns fast food consumption. We followed the lead of prior research [38] and created a measure of fast food consumption using an item from the second wave of data collection. This item inquired about the frequency of fast food consumption during the previous week. Examples of fast food were given during the interview, such as McDonald’s, Taco Bell, KFC, and Pizza Hut, in order to avoid misclassification. Response options for this item ranged from 0 (zero days during the previous week) to 7 (every day during the previous week).

*Low Vegetable Consumption.* In addition to our measure of fast food consumption, we created a second indicator of poor food quality that tapped infrequent vegetable consumption. During wave 2 of data collection, respondents were asked a number of specific questions regarding their eating habits. In particular, 12 questions were employed to determine whether respondents ate a number of different vegetables the day prior to the interview, including broccoli, carrots, kale, spinach, cabbage, squash, and green beans. We followed the lead of prior research [39] and utilized these questions in the creation of this item. Response options for each item were binary, including 0 (did not eat yesterday) and 1 (ate yesterday). The final measure was created so that all respondents who indicated that they did not eat any of the vegetables asked about in the questions were coded as a 1, whereas respondents who reported eating any of the vegetables during the previous day were coded as a 0.

*Meal Deprivation.* In addition to two measures of poor food quality, we created a measure of meal deprivation. In an effort to determine whether each subject was consuming an adequate amount of food, we created an item that measured how frequently subjects were failing to eat regular meals during their adolescent years. At wave 2, participants were asked three questions about the number of days during the past week in which they ate specific meals: one question about breakfast, one about lunch, and one about dinner. Each question had response options ranging from 0 (zero days) to 7 (seven days). Scores on these items were reverse coded so that higher scores indicated a greater tendency to skip that particular meal (e.g., breakfast). A composite measure was created in order to determine the overall extent of meal deprivation across all meals. This measure was created by summing together the scores on the reverse-coded items and then dividing by the total possible number of meals reported (*i.e.*, 21) in order to yield the proportion of meals that were not eaten during the previous week. Scores closer to 1 on this item indicate a greater degree of meal deprivation, whereas scores closer to zero indicate more regular consumption of meals.

#### 2.2.3. Physical Activity Measures

*Low Sports Involvement*. A number of questions regarding different kinds of physical fitness/activity were asked of adolescent respondents at the second wave of data collection, including how frequently the subject participated in an active sport during the week prior to the interview. Subjects were asked, “During the past week, how many times did you play an active sport, such as baseball, softball, basketball, soccer, swimming, or football”? Response options included not at all (0), 1 to 2 times (1), 3 to 4 times (2), and 5 or more times (3). The item was reverse-coded so that respondents who participated in sports less frequently received higher scores.

*Low Cycling/Skating*. A wave 2, respondents were also asked how many times during the past week they participated in roller-blading, roller-skating, skateboarding, or bicycling. Response options for this item also ranged from 0 (not at all) to 3 (5 or more times). The item was reverse-coded in order to reflect low levels of cycling/skating.

*Low General Exercise*. Finally, subjects were also asked, “During the past week, how many times did you exercise, such as jogging, walking, doing karate, jump roping, doing gymnastics or dancing”? Responses options were coded in the same manner as the previous physical activity measures.

*Insufficient Exercise*. In an effort to identify those participants who failed to engage in an adequate amount of exercise during the previous week, regardless of type, we created a general measure of insufficient exercise using the three exercise measures listed above. Following the lead of Ornelas, Perreira, and Ayala [40], we created a binary measure of insufficient exercise in which respondents who participated in less than five bouts of physical activity a week, regardless of type, were assigned a value of 1, whereas respondents who participated in five or more bouts of physical exercise a week, regardless of type, were assigned a value of 0. The cut-off point was used in an effort to approximate the recommended amount of exercise advocated by the American Heart Association as well as the Center for Disease Control and Prevention, which is that individuals participate in five exercise sessions a week, lasting at least 30 min (a total of 150 min or more). Although we could not ascertain the actual number of minutes of exercise each participant engaged in, the number of exercise sessions gives us a close approximation as to whether or not the subject is engaging in a sufficient or insufficient amount of physical activity.

### 2.3. Plan of Analysis

The analysis for the current study will be carried out using a technique known as Defries-Fulker (DF) analysis. DF analysis is a regression-based method that is capable of providing specific estimates of the relative effects of genetic factors, shared environmental factors, and nonshared environmental factors. These estimates are obtained by using samples of sibling pairs who differ in their degree of genetic similarity (e.g., MZ and same-sex DZ twins). DF analysis decomposes the variance in the outcome variable into the proportions explained by genetic and environmental factors, while also allowing for the estimation of regression coefficients for specified nonshared environments (*i.e.*, environments that are not shared by siblings within a kinship pair). We chose to employ DF analysis in order to ensure that any associations between nutritional factors, fitness factors and verbal intelligence are not spurious due to genetic influences. The current genetically informed analysis is an effort to determine whether the purported link between diet, physical activity and verbal intelligence is environmentally transmitted or spurious due to unmeasured genetic influences.

The DF equation has been revised since it was originally postulated by DeFries and Fulker [41,42] in order to be fit for use among samples drawn from the general population [43]. The revised equation is depicted as follows:

K_1_ = *b*_0_ + *b*_1_K_2_ + *b*_2_R + *b*_3_(R × K_2_) + *e* (1)

In this equation, K_1_ represents the PVT percentile score (*i.e.*, the outcome variable) for one of the twins being analyzed, K_2_ represents their co-twins PVT percentile score, R is an indicator of the genetic similarity between the kinship pair (1 for MZ twin pairs and 0.5 for DZ twin pairs), and R × K_2_ is an interaction term that multiplies the co-twin’s PVT percentile score by their degree of genetic similarity with their twin. Moreover, *b*_0_ represents the constant, *b*_1_ represents the proportion of the variance in verbal ability that is explained by shared environmental influences, *b*_2_ is not interpreted in the DF model, and *b*_3_ is the proportion of the variance in verbal intelligence that is explained by genetic influences. The error term (*e*) encompasses the effects of the nonshared environment on verbal intelligence and error.

Recently, Rodgers and Kohler [44] proposed an improvement to Equation (1) that only slightly alters its form. The new equation is depicted as follows:

K_1_ = *b*_0_ + *b*_1_(K_2_ – K_m_) + *b*_2_[R × (K_2_ − K_m_)] + *e* (2)

In this equation, K_1_, K_2_, R and *e* have the same significance as they do in Equation (1). However, this equation includes the term K_m_, which represents the mean value of K_2_ (or, in this study, the mean verbal intelligence score for the co-twins). Therefore, the parenthetical statement K_2_ – K_m_ signifies that K_2_ is mean-centered in this equation. Just as was the case in Equation (1), *b*_0_ represents the constant and *b*_1_ represents the proportion of the variance in verbal intelligence that is explained by shared environmental influences. However, in this updated equation, *b*_2_ (instead of *b*_3_) is interpreted as the proportion of the variance in verbal intelligence that is explained by genetic influences.

The coefficients in the above equation do not tell us the effect of any particular gene or shared environment on verbal intelligence precisely because the coefficients signify latent factors. Nevertheless, Equation (2) can be altered slightly to allow for the inclusion of specific nonshared environments of interest. In the current study, we make use of the following equation in order to examine a number of nonshared environments related to nutrition and exercise, in an effort to determine whether these factors have a significant influence on verbal intelligence, net of genetic and shared environmental factors. The DF equation that allows researchers to include specific nonshared sources of variance is depicted as follows:

K_1_ = *b*_0_ + *b*_1_(K_2_ – K_m_) + *b*_2_[R × (K_2_ − K_m_)] + *b*_3_ENVDIF + *e* (3)

Equation (3) is almost an exact replication of Equation (2). The only difference is the term ENVDIF. ENVDIF represents the difference score that is created when one twin’s score on a variable is subtracted from their co-twin’s score on the same variable. In the current study, difference scores are calculated for each of the nutrition and physical activity variables in order to determine if sibling differences in these variables predict differences in verbal intelligence, net of genetic and shared environmental influences. Importantly, *b*_3_ in Equation (3) does not represent a latent factor, but instead represents a regression coefficient, and needs to be interpreted as such (e.g., using critical *t*-values, *p*-values, *etc.*).

A series of DF models were estimated in the present study. The first model employs the baseline DF equation (Equation (2)) in order to ascertain the proportion of the variance in verbal intelligence that is due to genetic, shared environmental, and nonshared environmental factors. Subsequent models employ the formula displayed in Equation (3), which allows us to introduce the nutrition and exercise variables as nonshared sources of variance by including them as difference scores (ENVDIF) in the equation. The goal of these analyses is to determine whether differences between the twins in diet and physical activity significantly contribute to differences in their verbal scores, independent of genetic and shared environmental influences.

In order to maximize the information available on twin pairs in the Add Health, and in line with prior research [45,46,47], twins were double entered. Double-entering allows for each twin to be both the independent and dependent variables in the DF analysis. Despite this advantage, double entering violates the assumption of the independence of observations (since the same observations are repeated twice). Violation of this assumption results in deflated standard errors, which biases tests of statistical significance. We corrected for this in our study by employing Huber-White standard errors, which allows us to take account of the clustering of observations when estimating the statistical significance of the results.

## 3. Results

Table 2 displays the results of the DF models examining the influence of the shared environment, heritability, and adolescent nutritional factors on verbal intelligence during early adulthood. Model 1 of Table 2 contains the results of the baseline model, which identifies no specific nonshared sources of variance and only estimates the proportion of the variance in verbal intelligence due to heritability and the shared environment. The results of model 1 indicate that shared environmental factors explain approximately 44% of the variance in verbal intelligence during early adulthood, while 27% of the variance in verbal intelligence during early adulthood is due to genetic factors (in both cases, *p* ≤ 0.05). From these results, we can deduce that a combination of the nonshared environment and error account for the remaining 29% of the variance in verbal intelligence during early adulthood.

Although model 1 allows us to estimate the relative influence of genes, the shared environment, and the nonshared environment, it does not estimate the effects of any specific genetic or environmental source of variance. Models 2 through 5, however, extend model 1 by including one or more of the nutritional factors as nonshared sources of variance. Model 2 includes all three measures of poor/inadequate nutrition in the DF equation simultaneously, whereas Models 3 through 5 include only one nonshared source of variance at a time. The results across models 2 through 5, however, paint a similar picture: twins who do not practice adequate nutrition during adolescence evince significantly poorer verbal intelligence during adulthood, net of shared environmental and genetic influences. Specifically, fast food consumption, low vegetable consumption, and meal deprivation during adolescence all result in statistically significant reductions in verbal intelligence by adulthood, even after accounting for the effects of genes and the shared environment on verbal intelligence.

**Table 2:** DF analysis of the shared environment, heritability, and adolescent nutritional factors as predictors of verbal intelligence during early adulthood verbal intelligence (wave 3).

	Model 1		Model 2		Model 3		Model 4		Model 5	
	*b*	SE	*b*	SE	*b*	SE	*b*	SE	*b*	SE
*DF Analysis Components*										
Shared Environment	0.44 **	0.10	0.48 **	0.10	0.49 **	0.10	0.47 **	0.10	0.47 **	0.10
Heritability	0.27 *	0.11	0.25 *	0.11	0.22 *	0.11	0.24 *	0.11	0.25 *	0.11
*Nonshared Sources of Variance*										
Fast Food Consumption			−0.94 *	0.45	−0.80	0.46				
Low Vegetable Consumption			−4.34 **	1.57			−4.35 **	1.56		
Meal Deprivation			−7.46 *	3.87					−8.10 *	3.93
N	694		644		646		648		646	
R^2^	0.41		0.45		0.44		0.44		0.45	

* *p* ≤ 0.05, two-tailed; ** *p* ≤ 0.01, two-tailed.

The coefficients for the nonshared sources of variance can be interpreted just as OLS regression coefficients are interpreted, realizing, however, that the unit of analysis is twin pairs (not individuals). For example, in the current analysis, the coefficients represent the average increase in verbal intelligence (measured as a percentile score) for every one-unit increase in the nonshared source of variance, relative to one’s twin. In the case of low vegetable consumption, which is a binary variable, the results suggest that, within twin pairs, the twin with lower vegetable consumption tends to score lower on verbal intelligence during early adulthood. More specifically, in cases where twins within a pair are discordant in terms of their vegetable consumption during adolescence, the twin with lower vegetable consumption will, on average, score 4.34 percentile points lower on verbal intelligence during early adulthood relative to their co-twin, even after taking genes and the shared environment into account.

Similar results are obtained in the case of meal deprivation, where going from no meal deprivation to absolute meal deprivation is predicted to result in a 7.46 to 8.10 percentile-point decrease in verbal intelligence, net of genetic and shared environmental factors. Furthermore, model 2 predicts that adding an additional day of fast food consumption per week relative to a co-twin will result in an almost full percentile point drop in relative verbal intelligence. Thus, when taken as a whole, the results of the models displayed in table two suggest a significant influence of adolescent nutritional factors on adult verbal intelligence.

Table 3 is presented in the same format as Table 2, except for the inclusion of an additional model due to a greater number of nonshared sources of variance being examined. Model 1 represents the baseline model, whereas models 2 through 6 introduce nonshared sources of variance related to physical fitness during adolescence.

**Table 3:** DF analysis of the shared environment, heritability, and adolescent physical fitness as predictors of verbal intelligence during early adulthood verbal intelligence (wave 3).

	Model 1		Model 2		Model 3		Model 4		Model 5		Model 6	
	*b*	SE	*b*	SE	*b*	SE	*b*	SE	*b*	SE	*b*	SE
DF Analysis Components												
Shared Environment	0.44 **	0.10	0.48 **	0.10	0.48 **	0.10	0.48 **	0.10	0.48 **	0.10	0.48 **	0.10
Heritability	0.27 *	0.11	0.23 *	0.11	0.23 *	0.11	0.23 *	0.11	0.23 *	0.11	0.24 *	0.11
Nonshared Sources of Variance												
Low Sports Involvement			0.22	0.87	−0.82	0.77						
Low Cycling/Skating			1.57	0.95			0.87	0.95				
Low General Exercise			−0.49	0.87					−1.30	0.77		
Insufficient Exercise			−4.11 *	1.94							−3.72 **	1.51
N	694		648		648		648		646		648	
R^2^	0.41		0.44		0.44		0.44		0.45		0.44	

* *p* ≤ 0.05, two-tailed; ** *p* ≤ 0.01, two-tailed.

The results from Table 3 indicate that, although no specific category of adolescent physical activity significantly influenced adult verbal intelligence, youth who failed to engage in a sufficient amount of exercise evinced lower verbal intelligence scores during early adulthood. To be precise, models 2 and 6 suggest that, even after accounting for genetic and shared sources of variance, twins who failed to engage in sufficient exercise during adolescence scored between 3.72 and 4.11 percentile points lower on adult verbal intelligence than co-twins who engaged in regular exercise during adolescence. Nevertheless, incremental reductions in particular forms of physical activity (e.g., sports, cycling) did not appear to result in significantly lower verbal intelligence by early adulthood (A sensitivity analysis that included all nutrition and exercise variables in the same DF model was also conducted. The results revealed that both high fast food consumption and low vegetable consumption significantly reduce verbal ability scores during early adulthood. However, in this full model, none of the exercise items predicted verbal ability scores, suggesting that the effect of exercise on verbal ability does not remain once nutritional factors are accounted for).

## 4. Discussion

Behavioral patterns relating to physical activity and diet have been shown to influence various cognitive functions, including verbal intelligence [1,3,4,5,6]. Research exploring the influence of adolescent nutritional and exercise habits on subsequent verbal intelligence, however, has been very limited, for exceptions, see [11,16,20]. Moreover, even fewer studies have accounted for the possibility that genetic factors might explain, at least in part, the link between diet, exercise, and verbal skills, but see [29]. The main objective of the current study was to address these voids in the literature by testing whether various nutritional and exercise differences between co-twins during their adolescent years predict differences in their verbal intelligence during early adulthood, net of genetic and shared environmental influences. The results of our study revealed three key findings.

First, our results indicated that both shared environmental factors and genetic factors explain a significant portion of the variance in verbal intelligence. To be precise, approximately 44% of the variance in verbal intelligence during early adulthood was attributable to shared environmental factors, whereas 27% of the variance in verbal intelligence during early adulthood was attributable to genetic factors. These results also inform us that the remaining portion of the variance (*i.e.*, 29%) can be attributed to nonshared environmental influences and error. Prior research has often detected a higher degree of genetic influence on verbal ability as one progress from childhood to early adolescence [48]. In young adult samples, heritability typically remains quite high, which differs from the relatively modest estimate in the present study [49]. Nevertheless, the findings underscore the utility of genetically-informed designs in highlighting the relative contribution of genes, the shared environment, and the nonshared environment. Furthermore, the inclusion of the heritability and shared environment coefficients in subsequent models exploring the link between nutrition, physical activity, and verbal intelligence assures us that the significant associations detected are robust to both shared environmental and genetic influences.

Second, the results revealed that poor nutrition during adolescence corresponds to a significant decrease in verbal intelligence during early adulthood, even after accounting for genetic and shared environmental factors. In particular, when twins were discordant in their eating habits, the average verbal intelligence score for twins with poorer nutrition was significant lower than the verbal intelligence score of their cotwins. These results held across multiple measures of nutrition, including fast food consumption, low vegetable consumption, and meal deprivation.

Third, we found that youth who did not engage in a sufficient amount of exercise were more likely to display lower verbal intelligence during early adulthood. Specifically, relative to their physically active co-twins, twins who were more sedentary during adolescence showed inferior verbal intelligence during early adulthood. We found no evidence, however, that small reductions in the frequency of specific forms of physical activity (e.g., 3 days a week to 2 days a week) resulted in lower verbal intelligence during early adulthood. In the case of adolescent exercise, therefore, significant reductions in adult verbal intelligence do not seem to emerge until respondents drop below a particular threshold of physical activity (*i.e.*, five exercise sessions a week).

## 5. Conclusions

The current study is, to our knowledge, the first to provide a genetically informed test of the relationship between adolescent health behaviors (*i.e.*, diet and physical activity) and verbal intelligence during adulthood. Despite the unique contribution of the study, it is not without its limitations. First, we would have preferred the operationalization of our key independent variables to be more precise. For example, our measure of insufficient exercise identifies participants who failed to engage in five or more bouts of exercise per week. The exact content of each exercise session (e.g., intensity, length), however, is unknown. Such details would likely prove useful in determining the specific features of physical activity during adolescence that are most likely to impact subsequent verbal intelligence. Despite this shortcoming, the simplicity of the measurement of some of our key independent variables results in research implications that are straightforward and practical. For example, our findings regarding low vegetable consumption highlight a nutritional change that would be quite easy to implement, as they suggest that eating just one serving of vegetables a day during adolescence can lead to greater verbal intelligence during adulthood.

Second, a large body of research has linked diet and physical activity to a host of other cognitive functions apart from verbal intelligence, including perceptual organization, processing speed, memory and visual-spatial skills [1,27]. We would have preferred to have had access to a more comprehensive battery of cognitive assessments at wave 3 in order to test the robustness of our results. Nevertheless, such data were not available in the Add Health. Finally, our use of a sample of same-sex twin pairs may limit the generalizability of the results. We should note, however, that a recent study by Barnes and Boutwell [50] suggests that twin-based research may be more generalizable to the population of singletons than previously assumed.

In conclusion, while research has repeatedly demonstrated the impact of nutrition and physical activity on the verbal capacity of children and older adults, it appears that specific aspects of brain development and functioning are impacted by diet and exercise choices during the teenage years. The association between these health behaviors and verbal intelligence also seems to exist independent of genetic and shared environmental influences, suggesting that relatively simple changes to the diet and exercise regime during the adolescent years can have a significant influence on verbal intelligence during early adulthood. Future studies should explore the generalizability of these results to other aspects of cognitive functioning. Furthermore, replication of Luciano and colleagues’ [29] study is also needed to further elucidate the extent to which health behaviors and intelligence have shared genetic underpinnings. Finally, scholars should seek to employ more specific measures of nutrition and exercise (e.g., vitamin content of food, intensity/duration of exercise session) in an effort to build upon the results of the current study. Additional research that seeks to identify the specific dietary and exercise habits of youths that have the greatest impact on brain health will hopefully inform the practical behavioral changes during the adolescent years that can improve life quality during adulthood.
